# RNA Pseudouridylation in Physiology and Medicine: For Better and for Worse

**DOI:** 10.3390/genes8110301

**Published:** 2017-11-01

**Authors:** Marianna Penzo, Ania N. Guerrieri, Federico Zacchini, Davide Treré, Lorenzo Montanaro

**Affiliations:** Department of Experimental, Diagnostic and Specialty Medicine, Alma Mater Studiorum-University of Bologna, Via Massarenti 9, 40138 Bologna, Italy; marianna.penzo@unibo.it (M.P.); anianaila.guerrieri2@unibo.it (A.N.G.); federico.zacchini3@unibo.it (F.Z.); davide.trere@unibo.it (D.T.)

**Keywords:** RNA modification, pseudouridine synthase, dyskerin, non-coding RNA, gene expression control, cancer, inherited disorders

## Abstract

Pseudouridine is the most abundant modification found in RNA. Today, thanks to next-generation sequencing techniques used in the detection of RNA modifications, pseudouridylation sites have been described in most eukaryotic RNA classes. In the present review, we will first consider the available information on the functional roles of pseudouridine(s) in different RNA species. We will then focus on how alterations in the pseudouridylation process may be connected with a series of human pathologies, including inherited disorders, cancer, diabetes, and viral infections. Finally, we will discuss how the availability of novel technical approaches are likely to increase the knowledge in this field.

## 1. Introduction

A considerable number (>100) of different RNA modifications have been described [1]. Of these, pseudouridine (Ψ) is the most frequent and the first to be discovered —more than 50 years ago [2]. For these reasons it was named the “fifth RNA nucleotide” [3].

The presence of Ψ was first described in ribosomal RNA (rRNA) and transfer RNA (tRNA) [3], and subsequently in small nuclear RNAs (snRNA) [4,5,6]. More recently, thanks to the next-generation sequencing techniques applied for the detection of RNA modifications, updated databases have reported the presence of more than 9500 putative Ψ modification sites in most RNA classes of mammals and yeast—including mitochondrial tRNAs—(Mt-tRNAs), small Cajal Body-specific RNAs (scaRNAs), small nucleolar RNAs (snoRNAs), microRNAs (miRNAs), long intergenic non-coding RNAs (lincRNAs), miscellaneous other RNAs (misc_RNAs), and messenger RNAs (mRNAs) from both protein-encoding genes and pseudogenes [1,7]. Although only a limited number of these sites have been validated, the high number of putative sites suggests that pseudouridylation is ubiquitously distributed in RNAs.

A series of studies, conducted mainly in the past two decades, has provided important evidence concerning the functional consequence of changes in RNA pseudouridylation for both physiological and pathological status. This review focuses on these aspects of pseudouridylation, with particular attention to what has been reported in mammalian or human models, or both.

## 2. Biogenesis and Molecular Impact of Pseudouridine

**Figure 1:**
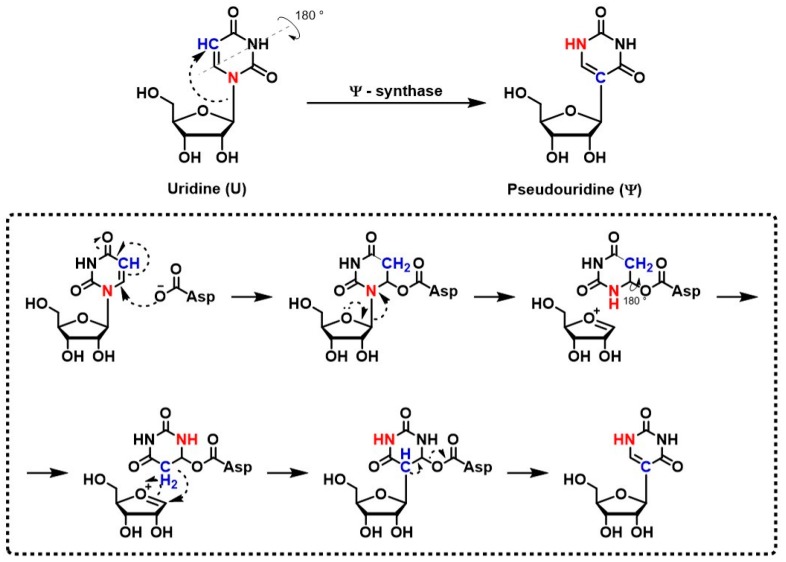
Example of the mechanism of Ψ/U conversion catalyzed by PUS1. For the sake of clarity some passages have been omitted.

Ψ in RNA derives from the C-C glycosidic isomerization of uridine, and the incorporation of C5 into the glycosidic bond (a scheme summarizing the reaction is shown in Figure 1); see [8,9] for review.

In RNA, uridine is transformed into pseudouridine by a class of enzymes known as pseudouridine synthases. From a structural standpoint, pseudouridine synthases, in both prokaryotes and eukaryotes, are generally classified into different families, five of which are named after *Escherichia coli* enzymes: TruD, TruA, TruB, RsuA (which is not present in eukaryotes) and RluA, and one (PUS10 family) is not found in *E. coli*. In eukaryotes, pseudouridine synthases can be either stand-alone enzymes (characterized by different substrates/modification sites and termed from PUS1 through PUS9 in yeast, and similarly, although with some differences, in humans—see Table 1) or part of RNA-guided ribo-nucleo-protein (RNP) complexes (in this case, the catalytic component is called dyskerin in humans, Cbf5 in yeast) (see [9] for review). For the reader’s convenience, Table 1 lists different yeast and human pseudouridine synthases.

Ψ in tRNA was among the first identified modifications [2,3]. Pseudouridylation of uridine residues in tRNAs is carried out by many of the pseudouridine synthases (PUSes) indicated in Table 1. With regard to tRNA Ψ modifications, yeast is by far the most well-studied organism among the eukaryotes. To date, different stand-alone PUSes have been identified as being responsible for pseudouridylation of specific uridine residues that may occur on all or some specific tRNAs, for a total of 15 sites. Most residues are pseudouridylated by PUS1 (residues at positions 26–28, 34–36, 65 and 67 [10]) although other enzymes also act at different residues, such as PUS3 (38, 39) [11], PUS4 (55) [12], PUS6 (31) [13], PUS7 (13, 35) [14], and PUS8 (32) [15]. Although there is still not enough experimental evidence in humans, the high homology between human and yeast PUSes suggests that human tRNAs may be modified by stand-alone enzymes similarly to what happens in yeast.

Eukaryotic rRNAs harbor 95 Ψs (equivalent to 1.4% of total rRNA nucleotides) which, although distributed quite uniformly over the lengths of 28S, 18S, 5.8S, and 5S, end up lying close to one another in the rRNA three-dimensional structure, once they are assembled with ribosomal proteins. In rRNA, uridine conversion to Ψ is carried out by enzymatic RNP complexes called boc H/ACA RNPs, each consisting of one H/ACA snoRNA and four core proteins (GAR1, NHP2, NOP10 and dyskerin, the latter being endowed with catalytic activity). H/ACA snoRNAs contain a conserved sequence called ´box H´ (standing for hinge) and an ACA box, so called because of the three nucleotides present at their 3′ end. H/ACA snoRNA are structurally characterized by the presence of a double hairpin, each harboring a pseudouridylation pocket, which is specific to a particular target sequence, based on base pairing. After the occurrence of the interaction between the guide RNA and the substrate, this is further stabilized by multiple interactions with small nucleolar ribonucleoproteins (snoRNPs) and dyskerin, which carries out the isomerization of the target uridine (for a broader review the reader is referred to [16,17]).

As previously mentioned, pseudouridylation also occurs in snRNA. In eukaryotic snRNA, a number of Ψs are present, and most of them, formation depends on RNA-guided RNP complexes [18]. 

As reported in the Modomics database (http://modomics.genesilico.pl) [1], pseudouridylation has also been described in different snoRNAs, but the functional role for most of these modifications has not yet been clarified.

Although pseudouridylation is more abundant in rRNAs, tRNAs, and other ncRNAs, its presence has recently also been described in mRNA [19,20,21]. Thanks to new transcriptome-wide mapping methods, it has been possible to identify the presence of Ψ in mRNA in a Ψ:U ratio of about 0.2–0.6%, which is much lower than in other RNA species [22].

Although a clear functional role of each pseudouridine in RNA is far from being demonstrated, it is known that Ψ, because of the presence of an extra hydrogen bond donor at its non-Watson-Crick edge (Figure 1), is characterized by chemical properties distinct from those of uridine and all other known nucleotides. In particular, the presence of Ψ is able to: (i) give greater rigidity to the phosphodiester backbone of the RNA; (ii) stabilize Ψ-A base pairs (compared to that of U-A base pairs) through some effects on base stacking and water coordination, thus affecting RNA structure, spatial conformation and, ultimately, its functional properties; at the same time; (iii) increasing the thermal stability (reviewed in [23,24]). As mentioned above, pseudouridines have been found in tRNA, rRNA, snRNA, snoRNA and scaRNA, and mRNA. Indeed, chemical and functional characteristics of Ψ itself significantly impacts target RNAs at different levels (from structure to function) by making base pairing easier and, ultimately, increasing duplex stability and influencing secondary structures [25].

## 3. Pseudouridine in Physiology

Currently, the functional importance of Ψ is well-accepted by the field-specific scientific community. This is due to its highly conserved but widespread localization within important functional RNA domains, and its abundance, and also due to increasingly plentiful evidence originating from different model organisms, ranging from bacteria through yeast to humans. Indeed, for most of the reported modifications, a functional role has been proposed.

The main role of pseudouridylation in tRNAs is to increase their stability, thanks to the described chemical properties of Ψ [25]. Ψs are found in all tRNAs, and generally localize in the anticodon stem-loop, in the D stem and, in a conserved fashion, in position 55 [26], thus contributing to the stabilization of its tertiary structure. In yeast, Ψs in positions 38 and 39 cause a misreading and frame shifting [27]. 

As for rRNA, Ψs are distributed in the rRNA sequence in such a way that, within the mature ribosome three dimensional (3D) structure, they concentrate mainly in the decoding site, mRNA channel, peptidyl transferase center, tRNA binding site, and ribosomal subunit interface (reviewed in [28]). In these locations, Ψs have been shown to be of crucial importance for the ribosome to assemble with the correct structure and for protein synthesis to take place properly [29,30,31,32].

There is indirect evidence that rRNA pseudouridylation may be important for the physiological maintenance of stemness: embryonic stem cells express high levels of rRNA pseudouridine synthase dyskerin, which has been found to regulate the expression of several stemness factors [33,34]. In addition, the impaired dyskerin function is the causative event of an inherited disease, characterized by the premature exhaustion of hematopoietic stem cell potential (see pseudouridine in disease section).

Although most investigations into the functional role of snRNA have been conducted in yeast, a few studies have been performed on human cells. U2 snRNA is the most extensively modified snRNA in humans: it contains more than 10 Ψ residues introduced by sequence-specific guide RNAs [35]. In human cell lines, the presence of Ψs in snRNA can affect the efficiency of pre-mRNA splicing, also through a cumulative effect on the formation of the early spliceosomal complex [36]. This particular function of Ψs may be related to their role in stabilizing the conformation of RNA [37]. These effects on the snRNA structure may be enhanced by the presence of multiple pseudouridine residues [38].

In yeast, another known site of pseudouridylation is within U6 snRNA, located in the spliceosome core and pseudouridylated by Pus1p during a particular growth phase known as the filamentous growth. Indeed, the absence of the Ψ in U6 snRNA leads to an arrest in filamentous growth [39]. Moreover, it has been observed that different U residues in yeast snRNA can be modified in an inducible manner by both stand-alone and RNA-guided pseudouridine synthases. These modifications are believed to be implicated in functional splicing under stress conditions [40,41].

Among ncRNAs, the telomerase RNA component (TERC), which has an H/ACA element, is also pseudouridylated by the pseudouridine synthase dyskerin. Thanks to the new methodology of pseudouridine sequencing (Ψ-seq), it has been demonstrated that TERC has two functionally important Ψ residues [19], one of which is located in a region involved in telomerase reverse transcriptase (TERT) binding and, consequently, with telomerase activity [42].

Pseudouridines were found in each mRNA portion, including the 3′ untranslated region (3′ UTR), coding sequence (CDS), and 5′ UTR regions, without specific preferences. Little is known about the functional role of mRNAs pseudouridylation. The presence of the modification seems to affect splicing and translation during in vitro assays. In fact, the presence of Ψs may increase mRNA half-life during heat shock stress [19], and is supposed to mediate a higher translation capability for some mRNAs [43]. Conversely, other data have shown that, in different conditions, pseudouridylation may repress in vitro translation [44]. In addition, experimental evidence obtained using artificial constructs indicates that pseudouridylation may also suppress translation termination by converting a nonsense codon into a sense codon, both in vitro and in vivo [45]. In this regard, computational analyses on crystal structures have predicted that a mismatch Ψ/U in a mRNA-tRNA base pair may produce non-canonical pairing in other codons, thus leading to an amino acid mis-incorporation [46]. Lastly, a study has shown how conversion of U into Ψ in two distinct sites of a pre-mRNA affects its splicing in *Xenopus* oocytes [47]. 

Altogether, these findings suggest that Ψ may have different effects on the stability of mRNA and translational control, thus adding another level of post-transcriptional regulation to gene expression. 

## 4. Pseudouridine in Disease

The importance of Ψs in physiological processes may be better appreciated by taking a look at the other side of the same coin: pathology. 

The first association ever reported between uridine modification and human disease was found in studies focusing on urinary metabolites in cancer patients [48,49]. Ψ, like other modified nucleosides, cannot be recycled and is eliminated with urine; Ψ levels in urine mostly depend on the glomerular filtration rate and RNA turnover, and therefore are very often found to be higher in cancer patients [50]. For this reason, the evaluation of urinary Ψs has been proposed as a potential tumor marker [51]. As is the case with many other proposed tumor markers, however, the assessment of urinary Ψ levels has never been included in routine diagnostics. 

Two of the best examples of a disorder associated with a pseudouridylation defect are X-linked Dyskeratosis Congenita (X-DC) and its more severe form, Hoyeraal-Hreidarsson syndrome; these are two rare inherited syndromes caused by mutations in DKC1, the gene encoding for the pseudouridine synthase dyskerin. X-DC was originally described by dermatologists by virtue of a peculiar muco-cutaneous triad: abnormal skin pigmentation, dystrophy of the nails, and leukoplakia of the oral mucosa [52]. However, life-threatening problems for X-DC patients are more often hematological in nature, since a progressive pancytopenia occurs in more than 90% of patients and represents the most frequent cause of death [53,54]. Initially, X-DC associated features have not been attributed to (at least in part) a pseudouridylation defect. In fact dyskerin, in association with the same proteins that participate in snoRNPs (GAR1, NHP2, and NOP10), binds to TERC, allowing its stabilization [55], and thus permitting telomerase activity. Initially, X-DC clinical manifestations were interpreted to be a consequence of reduced replicative potential and premature ageing caused by impaired telomerase activity alone. This theory was supported by the description of an autosomal dominant form of DC, caused by mutations in TERC [56]. However, a defect in rRNA pseudouridylation has been reported more recently in X-DC patients [19,40]; in addition, studies on mice with DKC1 mutations or perturbed expression have suggested that defects in ribosome biogenesis and/or pseudouridylation may contribute to the DC phenotype [57,58]. Moreover, it has been shown that the pseudouridine synthase activity of dyskerin is required for correct snoRNA expression and proper rRNA modifications, thus leading to accurate hematopoietic stem cell differentiation. In X-DC, dyskerin dysfunction leads to snoRNA perturbation, ultimately affecting hemopoiesis [59]. In addition to defects in proliferating tissues, one salient feature of X-DC is susceptibility to cancer. The rational link between dyskerin dysfunction and uncontrolled cell growth may, once again, be the pseudouridylation defect. Indeed, an increased tumor incidence has been observed in DKC1 hypomorphic mutant mice starting from the early generations, when telomeres are still very long, suggesting that the telomerase-independent dyskerin function may play an important role in promoting tumorigenesis [57]. In agreement with this conclusion, in cells from both X-DC patients and DKC1 hypomorphic mice, a selective defect has been demonstrated in the translation of a sub-group of cellular mRNAs containing the so-called internal ribosome entry site (IRES) elements [60]. IRES elements are nucleotide sequences in mRNA 5′ UTRs that mediate translation independently of other elements generally required for translation initiation [61]. In X-DC cells, the defect in IRES-mediated translation results in the relative impairment of the translation of specific mRNAs. Initially, the mRNAs encoding the anti-apoptotic factors Bcl-xL and XIAP, and the tumor suppressor p27, were identified as translational targets of defective dyskerin [60,62]. More recently, IRES-containing mRNAs encoding for the tumor suppressor p53 and the vascular endothelial growth factor (VEGF) have also been added to the list [63,64,65]. The observation that rRNA pseudouridylation is defective in the DKC1 hypomorphic mutant model supports the hypothesis that the intrinsic alteration of ribosome function is indeed involved in determining translational alterations and, in turn, the cancer susceptibility observed in X-DC. This hypothesis was also corroborated by observations performed in sporadic breast carcinomas, indicating that a sub-group of tumors characterized by low dyskerin expression also showed reduced rRNA pseudouridylation [66]. Dyskerin modification sites, indeed, are situated within specific domains of the ribosome, which are important for tRNA and mRNA binding; the reduction in modified uridine residues in the ribosome could result in impaired translation of specific mRNAs encoding for tumor suppressors, thus promoting neoplastic transformations [67,68]. Indeed, we demonstrated that ribosomes purified from DKC1 depleted cells are characterized by a reduction in global pseudouridylation, which is accompanied by intrinsic functional defects. In particular, hypo-pseudouridylated ribosomes showed an altered translation mediated by viral (CrPV) and cellular (p53, VEGF) IRES elements, and reduced translational fidelity [69]. These observations suggest that a reduced rRNA pseudouridylation may ultimately cause an imbalance in the translation of cancer contrasting (e.g., p53) or promoting (e.g., VEGF) factors.

In parallel to what has been found for dyskerin and pseudouridylation defects, we and other authors previously observed that, in human cancer, dyskerin expression and rRNA pseudouridylation levels may frequently be higher. In breast cancer, dyskerin expression levels and functions have been correlated with tumor progression and poor patient prognosis, with lower disease-free survival [66]. This scenario has been reported in many other human cancer types such as hepatocellular carcinomas [70], lung, and prostate cancers [71,72]. Notably, in lung cancer, the impact of dyskerin overexpression on the clinical outcome has been linked to its role in the maintenance of telomerase function [73]. Nevertheless, the molecular mechanisms underlying this phenomenon have not yet been clarified, and the literature is lacking in experimental studies involving dyskerin overexpression cellular models.

As described above, altered dyskerin pseudouridine synthase activity has been recognized as a potential trigger for cancer onset, in the case of both inherited syndrome-associated tumors and sporadic malignancies. It is not yet clear, however whether the altered dyskerin expression may be due to genetic mutations or to altered gene expression control. As far as the first hypothesis is concerned, a systematic analysis of the DKC1 gene sequence in mutational hotspots, in human sporadic cancers of different types, showed that DKC1 mutations are not a frequent event, and cannot be considered the cause of reduced dyskerin expression [74], thus suggesting that, in sporadic tumors, dyskerin expression may undergo some kind of (post-)transcriptional regulation. Indeed, dyskerin expression is closely related to the activity of c-MYC: DKC1 is a direct and conserved transcriptional target of c-MYC, and so far, this kind of interaction is the first strong correlation of dyskerin with an oncogene to be proven [75]. Worthy of note is the fact that increased c-MYC levels are not appreciable in all the cellular models of DKC1 overexpression, thus suggesting that also other factors may be involved in the control of dyskerin expression in human tumors [75]. Dyskerin function and regulation in cancer might also suggest that this protein may represent an interesting therapeutic target, at least in cancers where its expression levels are increased [76,77].

Recently, pseudouridylation has been implicated in other diseases, in addition to X-DC and cancer. Among these, Ψs have been recognized as regulators of viral latency processes in human immunodeficiency virus (HIV) infections [78]. In particular, it has been shown that the DKC1-H/ACA RNP complex is responsible for the pseudouridylation of 7SK ncRNA, which makes it possible to capture an HIV transcriptional co-factor called P-TEFb required for viral transcription elongation, thus preventing it from producing a complete HIV-1 transcript, which is necessary for viral replication [78]. 

Pseudouridylation has also been associated with the pathogenesis of maternally inherited diabetes and deafness (MIDD). In particular, a point mutation in a mitochondrial tRNA seems to prevent the pseudouridylation of one nucleotide, thus altering the tRNA tertiary structure. This may lead to higher tRNA instability, causing deficiencies in mitochondrial translation and respiration. Perturbations in respiratory patterns increase the production of reactive oxygen species that seem to affect more preferentially pancreatic beta cells, neurons, and hair cells in the cochlea, thus causing MIDD [79].

Mutations in *PUS* genes may be correlated to diseases, paralleling what occur in the case of DKC1. A known missense mutation in the *PUS1* gene has been described to cause a mitochondrial myopathy and sideroblastic anemia (MLASA) [80,81]. This mutation affects a highly conserved amino acid in the active site of the enzyme. Another nonsense mutation was reported in the N-terminal half of the protein, possibly leading to the synthesis of a shorter and inactive polypeptide [82]. These mutations would lead to impaired pseudouridylation of specific cytoplasmic and mitochondrial tRNAs, ultimately determining a perturbation in protein synthesis that can be involved in the pathogenesis of MLASA. In spite of this, most tissues are unaffected, possibly due to lower translational activity, or to the presence of some kind of tissue-specific compensatory mechanism [83]. Also, due to the pleiotropic effects of PUS1, the pathogenesis of MLASA may involve an impaired pseudouridylation of other RNA species.

In addition, a form of autosomal recessive mental retardation (also known as MRT55) is caused by a homozygous mutation in the *PUS3* gene [84]. Shaheen et al. presented a study of a multiplex consanguineous family in which a homozygous mutation in *PUS3* was demonstrated to cause an intellectual disorder. Notably, this phenotype was linked to the reduction of pseudouridylation in specific positions on tRNAs [84].

Finally, three different meta-analysis genome-wide association studies identified *PUS10* as a shared-risk locus in Chron’s disease ulcerative colitis and celiac diseases [85,86,87,88]. 

In sum, different pseudouridine synthases are causative or participant in the onset of numerous diseases, underlying the importance of their function in the maintenance of cellular homeostasis.

## 5. Perspectives

As can be seen from this review, the role of pseudouridylation in biological control processes is far from being defined, and should become the subject of many more studies. In fact, it is likely to play a very important role in various physiological and pathological contexts that may involve novel biological concepts such as ribosome diversity, tRNA mediated translational control, and dynamic RNA modification. In these contexts, pseudouridylation may represent an additional level of complexity in the regulation of gene expression, which has been only partially explored up to now.

To tackle these issues, the contribution of innovative technical approaches is needed. In this regard, various techniques appropriate for the study of the role of pseudouridylation in the control of gene expression, have become available over the past few years; these are summarized for the reader’s convenience in Table 2. 

During the 1980s, different techniques, spanning from liquid chromatography/mass spectrophotometry (LC/MS) to high performance liquid chromatography (HPLC) and capillary electrophoresis, were developed and optimized for Ψ identification (See Table 2).

In 1993, Bakin and Ofengand set up the first Ψ chemical detection method based on the use of the *N*-cyclohexyl-*N*′-beta-(4-methylmorpholinium) ethylcarbodimide (CMC) [89]. CMC has the property to bind both to U-like and G-like residues and then, after alkaline removal, to remain linked only to Ψ residues. The presence of CMC on the residues prevents reverse-transcription, giving rise to truncated complementary DNA (cDNA) products. Taking advantage of the CMC-based protocol, in the past few years, different high-throughput techniques with single nucleotide resolution have been developed for the identification of Ψ residues within the whole transcriptome. These approaches are Ψ-seq [19], Pseudo-seq [20], Pseudouridine Site Identification sequencing (PSI-seq) [21], and CeU-seq [22]. These techniques are very similar to one another, but each of them has differences with regard to library preparation, enrichment and isolation of Ψ-containing transcripts. The Ψ-seq method by Schwartz et al. [19] is the first high-throughput single-nucleotide resolution method of Ψ detection which has been reported in the literature. It is a semi-quantitative approach set up by coupling the CMC-based method [89] with the recently developed strand-specific RNA sequencing (RNA-seq). The Ψ-seq method was used to validate most of the previously reported sites in rRNA, tRNA and snRNA, and to identify some putative novel sites of pseudouridylation within snoRNAs and mRNAs. These results helped confirm the presence of Ψ residues in different types of RNAs, and their dynamic and inducible nature [19].

Of notice, the CeU-seq method by Li et al. [22] is based on a chemically synthesized CMC derivate that pre-enriches the Ψ-containing RNA through biotin pulldown. Using this approach (reportedly 20–40% more sensitive), Li et al. [22] revealed that Ψ residues are more frequent than expected, and that pseudouridylation dynamic behavior is associated with the exposure to specific stimuli [22]. 

In addition to these next generation sequencing (NGS)-based techniques, the CMC property of binding tightly to pseudouridines was also taken advantage of in mass spectrometry detection. In recent decades, the Limbach group developed and optimized different methods for solving the problem of “silent” psi identification in mass spectrometry [102]. The ligation of CMC indeed caused a gain of 252 Da, allowing the identification of peaks corresponding to pseudouridines. More recently, the same research group set up a new assay called Selected Reaction Monitoring (SRM), a semi-quantitative approach which uses synthetic oligonucleotides that may or may not contain pseudouridine, and compares the ion abundance of the pseudouridine-specific fragment ion with that of the pseudouridine-containing oligonucleotide found in the original sample [95]. 

The CMC is also used in in vitro assays, with the aim of registering pseudouridylation activity on synthetic rRNA substrates [77,103]. 

With regard to the application of CMC in Ψ identification, some caution should in any case be exerted, taking into account the reported inconstant efficiency of the CMC binding and cleavage to/from U and G residues [91]. This would require careful consideration of the reproducibility of the results in independent assays (particularly on semi-quantitative data and on data regarding the inducible nature of Ψ modifications).

The reported methods that have been recently made available are likely to increase the understanding of the role of RNA modifications, and their connections to the structure and the function of the modified RNA. In this sense, relevant information on the pathogenic mechanisms involved in most of the cited disorders has been obtained. This could hopefully pave the way to the identification of novel therapeutic approaches. 

## Figures and Tables

**Table 1:** Eukaryotic pseudouridine synthases.

Gene Name	Enzyme	Catalytic Domain Family	Localization/Predicted Localization	Substrate
*PUS1* (*MLASA1*) (y/h)	Pseudouridine synthase 1 tRNA pseudouridine synthase A	TruA	Nucleus	tRNA (c), snRNA, mRNA
*PUS2* (y)	Pseudouridine synthase 2	TruA	Mitochondria	tRNA (m)
*TRUB2* (h)	TruB pseudouridine synthase 2	TruB	Mitochondria	tRNA(m)
*PUS3* (y/h)	Pseudouridine synthase 3	TruA	Nucleus, Cytoplasm	tRNA (c/m), mRNA
*PUS4* (TruB1) (y/h)	Pseudouridine synthase 4	TruB	Nucleus, Mitochondria	mRNA
*PUS5* (y)	Pseudouridine synthase 5	RluA	Mitochondria	rRNA (m)
*RPUSD1* (h)	RNA Pseudouridine Synthase Domain Containing 1	RluA	?	?
*RPUSD2* (h)	RNA Pseudouridine Synthase Domain Containing 2	RluA	?	?
PUS6 (y)	Pseudouridine synthase 6	RluA	Cytoplasm, Mitochondria	tRNA (m) mRNA
*RPUSD3* (h)	RNA Pseudouridine Synthase Domain Containing 3	RluA	Mitochondria	rRNA (m)
*RPUSD4* (h)	RNA Pseudouridine Synthase Domain Containing 4	RluA	Mitochondria	rRNA (m)
*PUS7* (y/h)	Pseudouridine synthase 7	TruD	Nucleus, Cytoplasm	tRNA(c), mRNA
*PUS7L* (h)	Pseudouridine synthase 7 homolog-like protein	TruD	?	tRNA(c)
*PUS8 (RIB2)* (y)	Pseudouridine synthase 8	RluA	Cytoplasm	tRNA(c)
*PUS9* (y)	Pseudouridine synthase 9	RluA	Mitochondria, Nucleus, Cytoplasm	tRNA (m), mRNA
*PUS10* (h)	Pseudouridine synthase 10	Pus10	Nucleus, Cytoplasm	tRNA(c), ?
*DKC1* (h/y)	Dyskerin	TruB	Nucleus, Nuceolus.	rRNA (n), sno/scaRNA, snRNA

The catalytic domain family is named after the corresponding bacterial enzymes. When known, the RNA substrate is indicated. Information is derived from the available literature cited in this review and from Uniprot, the National Center for Biotechnology Information (NCBI) and *Saccaromyces* Genome Databases. y: yeast; h: human; c: cytoplasmic, m: mitochondrial, n: nuclear; tRNA: transfer RNA; mRNA: messenger RNA; rRNA: ribosomal RNA; snoRNA: small nucleolar RNA; scaRNA: small Cajal body RNA; snRNA; small nuclear RNA.

**Table 2:** Available methods for pseudouridylation activity analyses, pseudouridine (Ψ) detection and functional assays on highly purified ribosomes.

Application		Method	Resolution	High-Throughput	References
**Pseudouridylation activity**		CMC-based assays	Site-specific	No	[77,89,90,91]
	snRNAs	TLC-based	Single-nucleotide	No	[92]
	Global Ψ	LC/MS	Potentially site-specific	No	[93,94,95]
	Global Ψ, tRNAs	HPLC	Single-nucleotide	No	[66,96]
**Identification/Quantification of Ψs**	Global Ψ	Immunological, antibodies	N/A	No	[97,98]
	Global Ψ	High performance capillary zone electrophoresis	N/A	No	[99]
	snoRNAs (TERC), mRNAs, rRNAs,	Ψ-seq	Single-nucleotide	Yes	[19]
	mRNAs, rRNAs,	Pseudo-seq	Single-nucleotide	Yes	[20,100]
	mRNAs, rRNAs	Pseudouridine Site Identification sequencing (PSI-seq)	Single-nucleotide	Yes	[21]
	mRNAs, rRNAs	CeU-seq	Single-nucleotide	Yes	[22]
**Characterization of the functional effects on translation**		Fidelity, IRES/Cap dependent translational efficiency			[101]

For the reader’s convenience, references have been indicated. CMC: *N*-cyclohexyl-*N*′-beta-(4-methylmorpholinium) ethylcarbodiimide; TLC: thin layer chromatography; LC/MS: liquid chromatography coupled with mass spectrometry; HPLC: high performance liquid chromatography; IRES: internal ribosome entry site; N/A: Not available; TERC: telomerase RNA component; IRES: internal ribosome entry site.
